# Developing a novel e-cigarette regulatory and policy control scale: results from the European Union

**DOI:** 10.1080/09687637.2021.1959520

**Published:** 2021-08-12

**Authors:** Ajay Shah, John Britton, Ilze Bogdanovica

**Affiliations:** School of Medicine, University of Nottingham, Nottingham, United Kingdom

**Keywords:** E-cigarettes, scale, regulations, smoking cessation

## Abstract

**Background:**

E-cigarette regulations vary considerably between countries though a standard approach for comparing regulatory frameworks does not exist. Additionally, there is no evidence on whether regulatory variations are associated with differences in e-cigarette use and smoking cessation. This study aims to develop a regulatory scale to measure and compare e-cigarette regulations between European Union countries and investigate whether scores are associated with e-cigarette use and smoking cessation.

**Methods:**

Data from a range of sources, such as ECigIntelligence, were used to develop a scale and score countries in the respective areas of e-cigarette scale. We used correlation analysis to investigate association between scale scores and e-cigarettes market, e-cigarette prevalence and use of e-cigarettes for smoking cessation.

**Results:**

An e-cigarette scale consisting of 10 domains was developed. Our analysis suggests that higher e-cigarette scale scores were associated with significantly greater use of e-cigarettes within countries, and greater increases in the prevalence of former smoking smokers between 2014 and 2017.

**Conclusions:**

Whilst further work is needed to develop the scale in line with rapidly changing regulatory landscape and product development, the current findings suggest that countries that have implemented e-cigarette regulations might be more successful in obtaining public health gains such as increase in the proportion of former smokers compared to countries where e-cigarette market and sales is not regulated.

## Introduction

E-cigarettes have been available on European markets since 2007, and in 2014 in the European Union (EU) around 48.5 million people had ever used an e-cigarette (Farsalinos et al., 2016). While the overall prevalence of daily use of e-cigarettes among Europeans remains low at between 1% and 2.9% (Kapan et al., 2020), prevalence varies considerably across the EU countries with ever use ranging from around 5% in Portugal and Italy to about 20% in Latvia (Eurobarometer, 2017).

Although e-cigarettes are used predominantly by former or current smokers, there have been concerns that e-cigarettes may act as a gateway to smoking (Etter, 2018) and in some countries, strict regulations to limit the use and sales of e-cigarettes have been implemented. Globally, there have been attempts to develop recommendations and legislative measures to reduce any potential risks related to e-cigarette use. The World Health Organization (WHO) aims to prevent the initiation of e-cigarette use by children and non-smokers specifically by recommending enforcement of regulations on minimum age of purchase, restricting promotions, sponsorship and flavours which may have appeal to children. In addition, the WHO advocates regulation of product characteristics, for example by restricting concentrations of components with serious toxicological effects, prohibiting the use of health claims (in the absence of a medicines license) and protecting regulatory frameworks from tobacco industry influence (WHO, 2016). The US Food and Drug Administration (FDA) regulates the e-cigarettes through, but not limited to, restricting access to e-cigarettes to under 18-year olds by law and education, regularly conducting inspections on manufacturing facilities, requesting information on ingredients and mandating pre-market reviews (FDA, 2020). In Europe in 2014 the revised European Tobacco Products Directive (TPD) harmonised e-cigarette legislation across the EU countries, including legislative measures on safety and quality requirements, packaging and labelling, and monitoring and reporting (Council Directive 2014/40/EU, 2014). In some countries, for example Finland, restrictions that exceed TPD requirements have been implemented and include bans on flavours, prohibitions of marketing, displaying and distance selling (WHO, 2020). In other European countries such as the United Kingdom (UK), which is no longer an EU member state, legislative measures that are more supportive of e-cigarettes use for smoking cessation and harm reduction have been implemented (“E-cigarettes and vaping: Policy, regulation and guidance,” 2020). However, there is no evidence as to whether these differences in e-cigarette legislation across the European countries are associated with changes in smoking prevalence over time.

In 2005 the first Tobacco Control Scale that aimed to quantify and compare tobacco control policy implementation in European countries was published (Joosens & Raw, 2020). The scale has been widely used in research to monitor implementation of tobacco control policies (Feliu et al., 2020); through comparing scale scores over time tobacco control progress can be explored. The aim of this study was to develop and test an equivalent regulatory and policy control scale to quantify the implementation of e-cigarette policies and to investigate associations between e-cigarette scale scores and a range of variables including e-cigarette market size, prevalence of e-cigarette use and changes in the proportion of former smokers across the current 27 EU nations and the UK.

## Methods

### E-cigarette regulatory and policy control scale development

#### Scale development

For the development of the scale we partly adopted best practice approach for item development and scale development for developing and validating scales for health research (Boateng et al., 2018). Using the component domains of the Tobacco Control Scale as a template (Joosens & Raw, 2006), we used policy and regulatory frameworks recommended and/or implemented by organisations including the EU, the WHO and the US FDA to identify distinct policy domains that apply to e-cigarettes. The relevant domains that were included in the final scale were decided on through extensive discussion between authors. We drew on Article 20 of the EU TPD where specific measures include, but are not limited to, restricting e-cigarette tank sizes, nicotine strength, refill container sizes, mandating warning labels on the potential toxicity of e-cigarettes, limiting cross-border trading of e-cigarettes and imposing mandatory notifications if firms sell and trade e-cigarettes (Council Directive Directive 2014/40/EU, 2014). The broadly similar approach advocated by the WHO was also considered (WHO, 2016). In a recent report using a sample of 68 countries, age-restrictions on purchase, bans on use in public places and promotional restrictions were identified to be the most commonly applied e-cigarette policies (Kennedy et al., 2017).

#### Scale domains

The scale development first included a decision on domains that needed to be included in the scale that was based on the areas that were identified as requiring regulations in TPD, WHO reports or FDA approach. Within each domain, we decided on what aspects need to be included and came up with items that were covered in each domain. Although initially we attempted to construct more detailed scoring for each item similar to that for TCS we found a lack of evidence that would allow data-driven decisions on the relative importance of each item.

#### Item construction

We created 24 items that were dichotomous statements, with “yes” or “no” responses scoring one or zero points, respectively, and zero scores allocated when relevant information was not available. We then developed the scale iteratively by testing the statements on eight randomly selected countries: Bulgaria, Finland, France, Germany, Italy, Latvia, Romania and the UK. The aim of these iterations was to minimise statement ambiguity, ensuring they could be considered in different regulatory frameworks and that statements could be answered with “yes” or “no.” There scores were initially allocated by AS and then discussed with JB and IB. In cases where authors disagreed with the initial scores, disagreement was solved through extensive discussion based on the information available. However, as the statements were dichotomous the only disagreement observed was related to the domain describing competent national authority: what evidence is considered sufficient for national annual representative surveys which monitor e-cigarette usage and which authorities are considered competent.

### Data sources

Information on national approaches to e-cigarette regulation for 27 Member States of the EU and the UK was gathered from national authority websites, from the online resource provided by the Johns Hopkins Bloomberg School of Public Health Institute for Global Tobacco Control (Institute for Global Tobacco Control Johns Hopkins Bloomberg School of Public Health, 2020), and from regulatory information provided by a commercial provider, ECigIntelligence (ECigIntelligence, 2019). We also e-mailed a simple questionnaire, pertinent to the scale, to relevant national Competent Authorities to corroborate and gather information (see Supplement 1 for Authorities contacted). The questionnaire consisted of 24 dichotomous statements, with the opportunity to add further comments as required. Questions included covered all aspects of the e-cigarette policy, for example, sales restrictions, restrictions on flavours and tank sizes. The questionnaire was developed by AS and reviewed by IB and JB synchronously with the development of the scale. The questionnaire used is available on request from the authors.

We obtained data from 2019 on the size of the e-cigarette market size (in EUR) for all EU Member States (excluding Cyprus due to unavailability) and the UK, using a fixed exchange rate from 2019, from Euromonitor International Limited (UK) (Euromonitor, 2019). The data provider only produced e-cigarette market size data in native currencies for Bulgaria and Croatia, which we converted into EUR using the exchange rate for October 1st, 2019 obtained from Fxtop company (Fxtop, 2020). To adjust for differences in national population we estimated market size per 1000 people, using population estimates from provided in the Eurobarometer report.

We used data from the 2017 Eurobarometer report which included data from the two most recent Eurobarometer surveys (2014 and 2017) to obtain data on (1) prevalence of e-cigarette use, (2) use of e-cigarettes resulting in either reduced tobacco consumption or quitting smoking among current or former smokers who have at least tried an e-cigarette or similar device, (3) the proportion of people who have stopped tobacco smoking, and (4) the change in the proportion of former smokers between the two surveys (Eurobarometer, 2017).

### Data analysis

We generated an e-cigarette control scale score for each country and ranked these to create an individual country rank whereby higher scores indicated a more rigorous regulatory framework for e-cigarettes. We then analysed the relation between the e-cigarette control scale scores and the following variables (1) e-cigarette use prevalence; (2) population-adjusted market size; (3) proportion of former and current smokers who had at least tried an e-cigarette and have quit smoking or reduced consumption as a result; and (4) change in the prevalence of former smoking between 2014 and 2017 by means of scatterplots and Spearman rank-correlation coefficients (*r*_sp_). Additionally, we explored whether market size was associated with the proportion of former or current smokers reporting e-cigarettes being useful for quitting or consumption reduction purposes. The choice of the variables for correlation analysis with the scale scores was made based on our hypotheses that (a) in countries with more restrictive regulations e-cigarette use is less prevalent and (b) scores reflecting regulatory environment supportive of e-cigarettes will be associated with greater e-cigarette market size, more frequent use of these products for quitting and more rapid changes in quitting over time. Our significance level was set at 0.05. Correlation analyses and scatter plots were carried out using GraphPad Prism (version 8.4.3 for Mac, San Diego, USA) and Stata v16.

## Results

### Domains of the scale

The domains included in the scale, and the statements within them, are listed in Table 1.

**Table 1.** E-cigarette scale.

Regulatory domain Yes (1 point) or No (0 points) or unknown
Age restrictions
E-cigarettes and e-cigarette related products are strictly for sale and use subject to a minimum age requirement of 18 years old
Sales and distribution of e-cigarettes and its related products
E-cigarettes and e-cigarette related products are prohibited for sale in general retail outlets
E-cigarettes and e-cigarette related products are prohibited for sale in online outlets
E-cigarettes and e-cigarette related products are prohibited for sale in self-service outlets including vending machines
Restrictions on cross-border sales, promotion and advertising of e-cigarettes and e-cigarette related products
Product restrictions and requirements
Restrictions are applied to flavours of e-cigarettes and e-cigarette related products
Restrictions are applied to e-cigarette tank sizes
Restrictions are applied to e-liquid nicotine strength
Restrictions are applied to e-liquid refill container sizes
Child and tamper-proof packaging are required for e-cigarettes and e-cigarette related products
Labelling, product information and packaging
Warning labels are required on the packaging of e-cigarettes and e-cigarette related products including information on toxicity, addictiveness and health warnings
Plain packaging is required for all e-cigarettes and e-cigarette related products, without demonstrating any form of manufacturer-related branding
Advertising, promotion and sponsorship
Restrictions on the advertisement and promotion of e-cigarettes and e-cigarette related products
Restrictions on the sponsorship of e-cigarettes and e-cigarette related products
Restrictions which aim to impede potential promotion or advertisement of e-cigarettes and e-cigarette related products to children
Use of e-cigarettes in public settings
Restrictions are applied to vaping in public places
Vaping is prohibited within vehicles carrying children
Notification of authorities
Mandatory notification of the appropriate regulatory is required when placing an e-cigarette and e-cigarette related products on the market
E-cigarette regulatory classification
E-cigarettes can be regulated as medicinal or pharmaceutical products, with the provision of a relevant cessation health claim
E-cigarettes are regulated fully or in part as consumer products
E-cigarettes are regulated fully or in part as tobacco products, inclusive as either a traditional tobacco product or as an e-cigarette product
Relevant national competent authority, standards agency, or public health body position on e-cigarette use
Evidence of national annual representative surveys which monitor e-cigarette usage for a minimum of three years
Evidence of a national endorsement of e-cigarettes as a smoking cessation intervention
Taxation of e-cigarettes and related products
Excise duties, or other relevant taxes specific only to e-cigarettes and/or e-cigarette related products are applied

### E-cigarette regulatory control scale scores for EU countries and the UK

**Figure 1.**
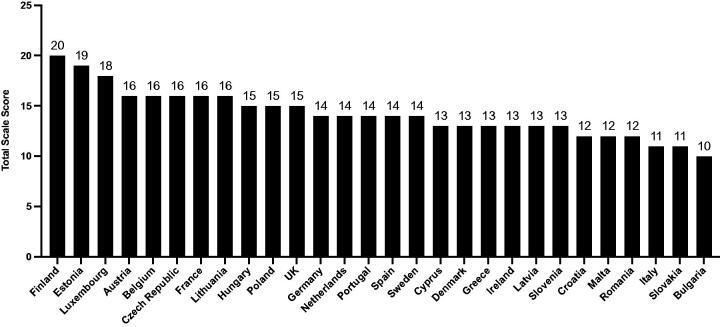
Scores of EU countries and the UK in rank order (highest to lowest scores from left to right).

We were able to obtain regulatory information from national Competent Authorities in Austria, Belgium, Czech Republic, Finland, France, Estonia, Lithuania, Luxembourg, the Netherlands, Poland, Spain and Sweden. For other countries, data were collected from sources available online as outlined above. National scores, with high scores indicating higher levels of regulatory restrictions on e-cigarettes, ranged from 10 to 20 out of a maximum of 24 points (Figure 1; full data provided in Supplement 2). Finland achieved the highest score (20), and Bulgaria the lowest (10). For 15 countries where responses form national Competent Authorities were not received, scores for the scale domain “Relevant national competent authority, standards agency, or public health body position on e-cigarette use” were not allocated and were included as zero in the total score.

### Relation between country scores and e-cigarette market size

**Figure 2.**
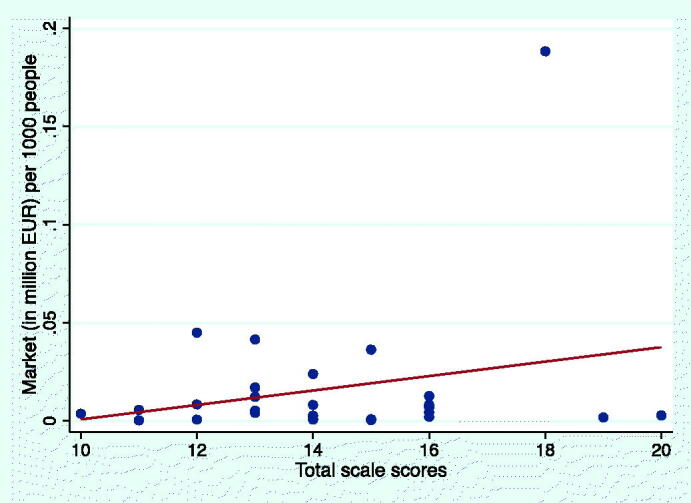
E-cigarette scale score and reported e-cigarette market size in EU member states and the UK. The plot was constructed using data from 27 countries (*r* = 0.01; *p* = 0.98).

In 2019, the two largest e-cigarette markets identified were the UK and France, valued at 2,417 and 847.1 million EUR, respectively. The smallest market was Slovakia, at 1.8 million EUR. Data for Cyprus were unavailable. There was no significant association between scale scores and estimated market size per 1000 population in the 27 countries for which data were available (Figure 2, *r* = 0.01, *p* = 0.98).

### Association between scores and prevalence of e-cigarette use

**Figure 3.**
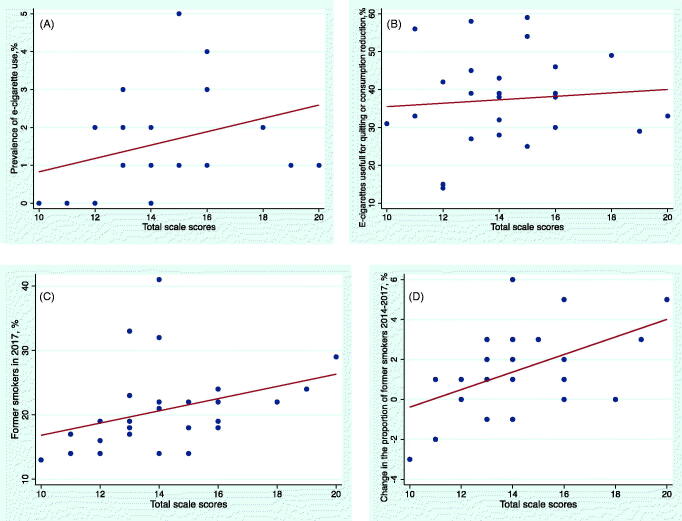
Scatter plot of e-cigarette scale score and reported survey response variables, including (A) prevalence of e-cigarette use (*r* = 0.4; *p* = 0.03). (B) the use of e-cigarettes resulting in quitting or consumption reduction (*r* = 0.12; *p* = 0.53), (C) proportion of former smokers in 2017 (*r* = 0.50; *p* = 0.007) and (D) the change in the proportion of people who have stopped smoking tobacco smoking between 2014 and 2017 (*r* = 0.45; *p* = 0.02).

The prevalence of current e-cigarette use in the countries studied ranged from 0% to 5%, with the highest prevalence occurring in the UK (5%). We observed a moderate positive association between scale scores and estimates of the prevalence of e-cigarette use across the 28 countries (Figure 3A; *r* = 0.40, *p* = 0.03). This suggests that in countries where e-cigarettes are more regulated, prevalence of e-cigarette use is higher.

### Association between the use of e-cigarettes for smoking cessation or reduction in tobacco consumption

**Figure 4.**
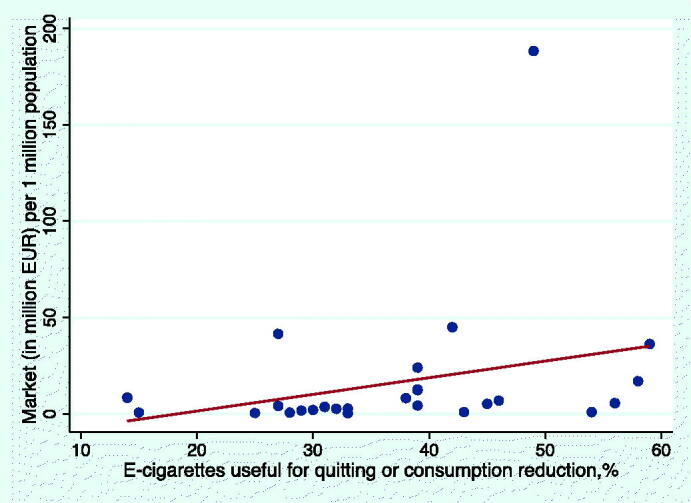
Association between the use of e-cigarettes resulting in quitting or consumption reduction and e-cigarette market adjusted for population size (*r* = 0.42; *p* = 0.03).

The proportion of former or current smokers who found e-cigarettes helpful for either quitting or reducing tobacco consumption varied from 14% in Croatia to 59% in the UK. There was no significant correlation between scale scores and the proportion of former or current smokers who had at least tried an e-cigarette and found that the use of e-cigarette (or similar device) had led to either quitting smoking or reducing tobacco consumption (Figure 3B; *r* = 0.12; *p* = 0.53). However, there was a significant association between population-adjusted market size and e-cigarettes being beneficial for reducing consumption or quitting smoking (Figure 4; *r* = 0.42; *p* = 0.03), indicating that in countries where more e-cigarette products are sold, a greater proportion of current or former smokers have successfully used e-cigarettes to quit or reduce tobacco consumption.

### Association between e-cigarette scale scores and the prevalence of ex-smokers

We found a significant association between e-cigarette control scale scores and the proportion of ex-smokers in national populations (Figure 3C; *r* = 0.50, *p* = 0.007) suggesting that in countries with more restrictive or comprehensive legislation on e-cigarettes there was a higher proportion of people who had successfully quit smoking. This was also true of the association with change in the proportion of former smokers between 2014 and 2017 (Figure 3D; *r* = 0.45; *p* = 0.02), suggesting that in countries where more regulations were in place a larger increase in the proportion of former smokers has been observed between 2014 and 2017.

## Discussion

### Summary of main findings

To our knowledge, this is the first study to develop a simple scale for e-cigarette regulation that can be used to compare policy implementation and enforcement measures between countries and identify regulatory areas that can be improved. We found that higher e-cigarette scale scores were associated with significantly greater use of e-cigarettes within countries, and greater increases in the prevalence of former smoking between 2014 and 2017. This suggests that countries with more regulation of e-cigarette sales, advertising and safety might be more successful in promoting quitting and reducing harm from tobacco smoking. The causal direction of this association is unknown however, with possibilities including greater regulation being a response to, rather than a cause of more widespread use, or use increasing despite or even because of greater regulation. Further longitudinal data will be required to begin to clarify this uncertainty.

### Comparison with previous research

Tobacco Control Scale scores have been widely used in research to monitor progress in tobacco control over time and compare it between countries and to explore regulations in relation to changes in smoking behaviour (Feliu et al., 2020; So et al., 2019; Serrano-Alarcon et al., 2019). The Tobacco Control Scale has been adopted for use in other contexts (Feliu et al., 2020, Movsisyan and Connolly, 2014). Whilst there have been studies exploring determinants of e-cigarette use between countries (Vardavas et al., 2015) we are not aware of any attempts to develop a scale that would allow exploration of changes in e-cigarette regulations and relations of those regulations with smoking behaviour in a similar way. The scale we present has therefore been created to offer a systematic approach for assessing national e-cigarette regulations and comparing progress and results between countries. International comparisons in regulations, particularly in relation to changes in smoking or e-cigarette use, are essential for global and national policy development to help demonstrate which approach works best to reduce public health harms associated with tobacco use.

### Strengths and limitations

Our scale was based on the well-established Tobacco Control Scale (Feliu et al., 2020) and similar to that scale we found low variance between countries, and measured policy existence rather than compliance or enforcement. It is likely that enforcement of many aspects of policy, such as sale restrictions, is difficult given the ease of online purchasing, as has proved to be the case in Australia, where e-cigarettes are banned (Douglas et al., 2015). Due to the EU TPD, in many countries the minimum level of restrictions is in place making it even harder to differentiate the scores between the 28 countries considered in this analysis. Wider scale analysis including a range of countries with more variation in e-cigarette regulations would provide better understanding of how well the scale captures implementation and enforcement of e-cigarette regulations. While the scale would benefit from more detailed scoring (instead of 0 or 1) the current evidence base did not allow us to create scores that would capture the relative importance of various policy elements. For example, whilst it is widely known that the price increases are the most effective tobacco control measure and that higher tobacco taxes benefit public health (Chaloupka et al., 2012) there is no evidence on whether higher taxation of e-cigarettes would be beneficial by reducing use among non-smokers, or harmful by discouraging switching among established smokers. A similar principle applies to other e-cigarette policy domains such as the product or sales restrictions. With a growing evidence base it should prove possible in the future to develop the scale to include relative comparisons of various legislation alternatives. It appears that in its current version the scale does not measure the strictness of regulations, only their presence. Another potential limiting factor was that the numbers used for the analysis in some cases were very low and did not differ between countries (e.g. e-cigarette use prevalence was 0% in several countries) making the detection of associations difficult. This could be addressed by using national level data which often provide more accurate estimates based on larger sample sizes (Bogdanovica et al., 2011).

### Conclusions and recommendations

This is the first attempt to develop a new scale that could provide valuable insight into international comparisons of quantified differences in e-cigarette regulations. Our findings using this scale suggest that in a set of countries with similar baseline e-cigarette regulations, higher scores are associated with greater proportion of population using e-cigarettes and a greater increase in the proportion of former smokers in the recent years prior to 2017 Eurobarometer survey. It is, therefore, possible that a regulated e-cigarette market can be beneficial for harm reduction. For policy makers the use of this scale will afford (a) comparison of the regulatory environment with other countries and (b) decide upon further regulations to reduce the health burden caused by smoking. Further development of this scale is needed in line with a growing evidence base. However, the current findings suggest that countries that have implemented e-cigarette regulations might actually be more successful in obtaining public health gains such as an increase in the proportion of former smokers compared to countries where e-cigarette market and sales is not appropriately regulated.

## Supplementary Material

Supplemental Material
