# Supplementary material for: Developing a novel e-cigarette regulatory and policy control scale: results from the European Union
Source: Drugs (Abingdon Engl). 2021 Aug 12;29(6):719–25. doi: 10.1080/09687637.2021.1959520 (PMC9721402; doi:10.1080/09687637.2021.1959520)
Supplement: Supplemental Material [file IDEP_A_1959520_SM5859.pdf]

Online supplement

Supplement 1 | Relevant national competent authority contacted

Member States	National Competent Authorities
**Austria**	Federal Ministry of Health
**Belgium**	Directorate General Animal, Plants, Food
**Bulgaria**	Ministry of Economy
**Cyprus**	Ministry of Health
**Czech Republic**	Ministry of Health
**Croatia**	Ministry of Health
**Denmark**	Danish Safety Technology Authority
**Estonia**	Estonian Health Board
**Finland**	National Supervisory Authority for Welfare and Health
**Germany**	Federal Office of Consumer Protection and Food Security
**Greece**	Ministry of Health
**Hungary**	National Institute of Pharmacy and Nutrition
**Ireland**	Health Service Executive
**Italy**	Ministry of Health, General Directorate of Prevention
**Latvia**	Health Inspectorate
**Lithuania**	Drug, Tobacco and Alcohol Control Department
**Luxembourg**	Ministry of Health
**Malta**	Ministry for Health
**Poland**	Inspector for Chemical Substances
**Portugal**	General Directorate of Health
**Romania**	Ministry of Health
**Slovakia**	Public Health Authority
**Slovenia**	National Laboratory for Health, Environment and Food
**Spain**	Ministry of Health, Social Services and Equality
**Sweden**	Public Health Agency of Sweden
**The Netherlands**	National Institute for Public Health and the Environment
**United Kingdom**	Medicines Healthcare Products Regulatory Agency

Supplement 2. Full dataset of responses. 0 corresponds to no, and 1 corresponds to yes; blank space corresponds to information missing.

	Age restrictions	Sales and distribution of e-cigarettes and its related products				Product restrictions and requirements					Labelling, product information and packaging		Advertising, promotion and sponsorship			Use of e-cigarettes in public settings		Notification of authorities	E-cigaerette product classification			Relevant national competent authority, standards agency, or public health body position on e-cigarette use		Taxation of e-cigarettes and related products	
	E-cigarettes and e-cigarette related products are strictly for sale and use subject to a minimum age requirement of 18 years old	E-cigarettes and e-cigarette related products are prohibited for sale in general retail outlets	E-cigarettes and e-cigarette related products are prohibited for sale in online outlets	E-cigarettes and e-cigarette related products are prohibited for sale in self-service outlets including vending machines	Restrictions on cross-border sales, promotion and advertising of e-cigarettes and e-cigarette related products	Restrictions on flavours of e-cigarettes and e-cigarette related products	Restrictions on e-cigarette tank sizes	Restrictions on e-liquid nicotine strength	Restrictions on e-liquid refill container sizes	Child and tamper-proof packaging are required for e-cigarettes and e-cigarette related products	Warning labels are required on the packaging of e-cigarettes and e-cigarette related products including information on toxicity, addictiveness and health warnings	Plain packaging is required for all e-cigarettes and e-cigarette related products, without demonstrating any form of manufacturer-related branding	Restrictions on the advertisement and promotion of e-cigarettes and e-cigarette related products	Restrictions on the sponsorship of e-cigarettes and e-cigarette related products	Restrictions which aim to impede potential promotion or advertisement of e-cigarettes and e-cigarette related products to children	Restrictions on vaping in public places	Vaping is prohibited within vehicles carrying children	Mandatory 0tification of the appropriate regulatory agencies when placing an e-cigarette and e-cigarette related products are placed upon the market	E-cigarettes can be regulated as medicinal or pharmaceutical products, with the provision of a relevant cessation health claim	E-cigarettes are regulated as consumer products	E-cigarettes are regulated as tobacco products	Evidence of national annual representative surveys which monitor e-cigarette usage for a minimum of three years	Evidence of a national endorsement of e-cigarettes as a smoking cessation intervention	Excise duties, or other relevant taxes specific only to e-cigarettes and/or e-cigarette related products applied	Total score
Austria	1	0	1	0	1	1	1	1	1	1	1	0	1	1	1	1	1	1	1	0	0	0	0	0	16
Belgium	1	0	1	0	1	0	1	1	1	1	1	0	1	1	0	1	1	1	1	1	1	0	0	0	16
Bulgaria	1	0	0	0	1	0	1	1	1	1	1	0	1	0	0	0	0	1	0	0	1			0	10
Croatia	1	0	0	0	1	0	1	1	1	1	1	0	1	1	0	1	0	1	0	0	1			0	12
Cyprus	1	0	0	0	1	0	1	1	1	1	1	0	1	1	0	1	0	1	0	0	1			1	13
Czech Republic	1	0	0	0	1		1	0	1	1	1	1	1	0	1	1	1	1	0	1	1	0	1	1	16
Denmark	1	0	0	0	0	0	1	1	1	1	1	1	1	1	0	1	0	1	1	0	1		0	0	13
Estonia	1	0	1	1	1	1	1	1	1	1	1	0	1	1	1	1	1	1	0	0	1	0	1	1	19
Finland	1	0	1	1	1	1	1	1	1	1	1	0	1	1	1	1	0	1	1	1	1	1	0	1	20
France	1	0	0	1	0	1	1	1	1	1	1	0	1	1	1	1	0	1	1	1	0	1	0	0	16
Germany	1	0	0	1	0	0	1	1	1	1	1	0	1	1	1	1	0	1	0	1	1			0	14
Greece	1	0	0	0	1	0	1	1	1	1	1	0	1	1	0	1	0	1	0	1	1			0	13
Hungary	1	0	0	1	1	0	1	1	1	1	1	0	1	1	0	1	0	1	0	1	1			1	15
Ireland	1	0	0	0	0	0	1	1	1	1	1	0	1	1	1	0	0	1	1	1	1			0	13
Italy	1	0	0	0	1	0	1	1	1	1	1	0	1	0	0	0	0	1	0	0	1			1	11
Latvia	1	0	0	0	1	0	1	1	1	1	1	0	1	1	0	1	0	1	0	0	1			1	13
Lithuania	1	0	1	0	1	0	1	1	1	1	1	0	1	1	1	1	1	1	0	0	0	1	0	1	16
Luxembourg	1	0	1	0	1	1	1	1	1	1	1	0	1	1	0	1	1	1	1	0	1	1	0	1	18
Malta	1	0	0	0	0	0	1	1	1	1	1	0	1	1	0	1	1	1	0	0	1			0	12
Netherlands	1	0	0	0	1	0	1	1	1	1	1	0	1	1	1	0	0	1	1	0	1	1	0	0	14
Poland	1	0	1	1	1	0	1	1	1	1	1	0	1	1	0	1	0	1	0	0	1	0	0	1	15
Portugal	1	0	0	1	1	0	1	1	1	1	1	0	1	1	0	1	0	1	0	0	1			1	14
Romania	1	0	0	0	1	0	1	1	1	1	1	0	1	1	0	1	0	1	0	0	1			0	12
Slovakia	1	0	0	0	0	0	1	1	1	1	1	0	1	1	0	1	0	1	0	0	1			0	11
Slovenia	1	0	1	0	1	0	1	1	1	1	1	0	1	1	0	1	0	1	0	0	1			0	13
Spain	1	0	0	0	1	0	1	1	1	1	1	0	1	1	1	1	0	1	1	0	1	0	0	0	14
Sweden	1	0	0	0	1	0	1	1	1	1	1	0	1	1	0	1	0	1	1	1	0	0	0	1	14
UK	1	0	0	0	0	0	1	1	1	1	1	0	1	0	0	1	0	1	1	1	1	1	1	1	15
